# Discoidin Domain Receptor 2 Contributes to Breast Cancer Progression and Chemoresistance by Interacting with Collagen Type I

**DOI:** 10.3390/cancers16244285

**Published:** 2024-12-23

**Authors:** Ai Sato, Kiyoshi Takagi, Momoka Yoshida, Mio Yamaguchi-Tanaka, Mikoto Sagehashi, Yasuhiro Miki, Minoru Miyashita, Takashi Suzuki

**Affiliations:** 1 Department of Pathology and Histotechnology, Tohoku University Graduate School of Medicine, Sendai 980-8575, Japan; ai.sato.b7@tohoku.ac.jp (A.S.); takashi.suzuki.c1@tohoku.ac.jp (T.S.); 2 Personalized Medicine Center, Tohoku University Hospital, Sendai 980-8574, Japan; 3 Department of Anatomic Pathology, Tohoku University Graduate School of Medicine, Sendai 980-8575, Japan; 4 Department of Breast and Endocrine Surgical Oncology, Tohoku University Graduate School of Medicine, Sendai 980-8575, Japan; minoru.miyashita.c6@tohoku.ac.jp; 5 Department of Pathology, Tohoku University Hospital, Sendai 980-8574, Japan

**Keywords:** discoidin domain receptor 2 (DDR2), collagen, breast cancer, chemoresistance, immunohistochemistry

## Abstract

**Background**: Chemoresistance is an important issue to be solved in breast cancer. It is well known that the content and morphology of collagens in tumor tissues are drastically altered following chemotherapy, and discoidin domain receptor 2 (DDR2) is a unique type of receptor tyrosine kinase (RTK). This RTK is activated by collagens, playing important roles in human malignancies. However, the contribution to the chemoresistance of DDR2 in terms of the association with collagens remains largely unclear in breast cancer. **Methods**: We immunolocalized DDR2 and collagen type I in 224 breast cancer tissues and subsequently conducted in vitro studies to confirm the role of DDR2 in breast cancer chemoresistance using chemosensitive and chemoresistant cell lines. **Results**: DDR2 immunoreactivity was positively correlated with aggressive behaviors of breast cancer and was significantly associated with an increased risk of recurrence, especially in those who received chemotherapy. Moreover, in vitro experiments demonstrated that DDR2 promoted the proliferative activity of breast cancer cells, and cell viability after epirubicin treatment was significantly maintained by DDR2 in a collagen I-dependent manner. **Conclusions**: These data suggested that DDR2 could be a poor prognostic factor associated with cell proliferation and chemotherapy resistance in human breast cancer.

## Simple Summary

Resistance to chemotherapy is an important issue in breast cancer, and it is closely associated with collagen remodeling. Discoidin domain receptor 2 (DDR2) is activated by collagens and plays important roles in human breast cancer. Here, we demonstrated the involvement of the DDR2/collagen type I axis in breast cancer progression and chemoresistance. DDR2 could be a poor prognostic factor in breast cancer patients.

## 1. Introduction

Breast cancer is one of the most prevalent cancers among women, and the estimated age-standardized incidence rate in 2022 was the first (46.8%) and the leading cause of cancer-related mortality was the second (12.7%) following lung cancer (16.8%) in the world [1]. Cytotoxic chemotherapy is used not only for estrogen-dependent breast cancer with a relatively aggressive phenotype but also for triple-negative breast cancer as a predominant therapeutic option. However, resistance to chemotherapy (chemoresistance) is considered a crucial issue in breast cancer and approximately 20% of patients do not respond to chemotherapy [2]. Therefore, understanding the mechanisms underlying chemoresistance is crucial to improve breast cancer treatment.

It is well known that the tumor microenvironment (TME) is closely related to malignant progression and that the interaction between cancer cells and stromal components contributes to proliferation, invasion, metastasis, and chemoresistance [3]. The TME consists of cellular components and the extracellular matrix (ECM), such as collagen, hyaluronan, and proteoglycan. Interestingly, the adhesion of cancer cells to the ECM triggers intracellular signaling to make the cancer cells resistant to drug resistance (so-called “cell adhesion-mediated resistance (CAM-DR)” [4]).

Collagen is the main structural component of the ECM in the human body. According to the difference in the structure, more than 29 kinds of collagens have been identified [5]. Among them, collagen type I is the most abundant form in the human body and is deposited in human cancer tissues, affecting tumor cell activity by interacting with its receptors [6]. Discoidin domain receptors (DDRs), DDR1 and DDR2, are a unique type of receptor tyrosine kinase (RTK) and are activated by collagens. Unlike other RTKs, DDRs undergo receptor phosphorylation in a sustained and delayed manner and initiate downstream signaling including regulating collagen production and degradation [7]. Both DDR1 and DDR2 can bind to collagen type I [8] and have been implicated in human malignancies [9]. However, it has been interestingly reported that the expression of DDR2 is elevated in breast cancer tissues compared to normal breast tissues, whereas that of DDR1 is decreased in breast cancer tissues [10]. In addition, accumulating studies have indicated the protumor roles of DDR2 in breast cancer [11,12]. It has been also interestingly reported that DDR2 regulates the stiffness of breast cancer tissues via collagen type I reorganization to induce lung metastasis [13].

The relationship between the DDR2/collagen axis and therapeutic resistance has also been reported. For instance, DDR2 expression is upregulated in oxaliplatin-resistant hepatocellular carcinoma cell lines and induces chemoresistance by inducing C-C motif chemokine ligand 20 (CCL20) via STAT3 activation [14]. In the clinical specimens, DDR2 expression has been demonstrated to be increased in chemoresistant epithelial ovarian cancers compared to chemosensitive ones [15]. DDR2 has also been demonstrated to contribute to tyrosine kinase inhibitors such as dasatinib [16] and sorafenib [17], and a combination of anti-BRAF and anti-MEK therapy (vemurafenib and cobimetinib, or dabrafenib and trametinib) [18]. On the other hand, collagen type I deposition inhibits drug uptake [19] and contributes to chemoresistance by interacting with integrin β1 in breast cancer [20]. Angiogenesis is closely associated with chemoresistance [21] and collagen type I contributes to angiogenesis to induce chemoresistance [22,23]. In addition, an antiangiogenic drug such as regorafenib also targets DDR2 [24]. However, the possible involvement of the DDR2/collagen type I axis in breast cancer chemoresistance has not been elucidated. In the present study, we therefore immunolocalized DDR2 and collagen type I in 224 invasive breast carcinoma tissues to clarify the biological and/or clinical significance and subsequently conducted in vitro studies to confirm the role of DDR2 in breast cancer chemoresistance using both chemosensitive and chemoresistant cell lines.

## 2. Materials and Methods

### 2.1. Patients and Tissues

A total of 224 specimens of invasive ductal carcinoma of the breast were obtained from Japanese female patients (age range 27–88 years) who underwent surgical treatment from 2005 to 2008 at Tohoku University Hospital (Sendai, Japan). All the specimens had been fixed in 10% neutral buffered formalin and embedded in paraffin wax. The clinical outcomes of the patients were evaluated by the disease-free survival and the breast cancer-specific survival, and the median follow-up time was 69 (range 2–136) months and 71 (2–136) months, respectively. The disease-free survival was defined as the time from surgery to recurrence and the breast cancer-specific survival was defined as the time from surgery to death from breast cancer. The research protocol was approved by institutional review boards and the ethics committee at Tohoku University School of Medicine (approval number 2023-1-457).

### 2.2. Immunohistochemistry

The rabbit polyclonal antibody against DDR2 was purchased from GeneTex (Irvine, CA, USA). The mouse monoclonal antibody against collagen type I was purchased from Proteintech (Rosemont, IL, USA). Immunohistochemistry was carried out using Histofine Simple Stain MAX PO (MULTI) kit (Nichirei Biosciences, Tokyo, Japan). The antigen–antibody complex was visualized with a 3,3′-diaminobenzidine (DAB) solution and subsequently counterstained with hematoxylin [25]. Ready-stained immunohistochemical preparations for ER, PR, HER2, and Ki67 were available from Tohoku University Hospital. In more detail, immunostaining of ER (CONFIRM anti-ER (SP1); Roche Diagnostics Japan, Tokyo, Japan) and PR (CONFIRM anti-PR (1E2), Roche Diagnostics Japan) was performed using Ventana Benchmark XT (Roche Diagnostics Japan), and that for HER2 was performed using HercepTest (DAKO, Carpinteria, CA, USA).

### 2.3. Scoring of Immunohistochemistry

DDR2 immunoreactivity was detected in the membrane and the cytoplasm of carcinoma cells. The cases with immunoreactivity of more than 20% positive area were considered positive for DDR2 according to the previous report [26]. Collagen type I immunoreactivity was detected in the stroma, and the cases with an immunoreactive area of more than 10% were considered positive [27]. ER, PR, and Ki67 immunoreactivity was detected in the nucleus of carcinoma cells, and the percentages of immunoreactivity (labeling index: LI) of more than 1% were considered positive for ER and PR [28,29]. HER2 immunoreactivity was evaluated according to the criteria of HercepTest (DAKO) (score 0–3) and the cases with a score of 3 were considered positive for HER2. For the cases with a score of 2, gene amplification was investigated by fluorescence in situ hybridization (FISH).

### 2.4. Cell Line and Chemicals

The human breast cancer cell line MCF-7 was obtained from the Japanese Collection of Research Bioresources Cell Bank (Osaka, Japan), and MDA-MB-231 and T47D were obtained from the American Type Culture Collection (ATCC; Manassas, VA, USA). These cells were cultured in RPMI-1640 (Fujifilm Wako Chemicals, Osaka, Japan) containing 10% fetal bovine serum (Gibco) and penicillin–streptomycin (Fujifilm Wako Chemicals).

Epirubicin (EPI)-resistant MCF-7 and MDA-MB-231 cells (namely M-EPIR and 231-EPIR, respectively) have been previously established [30]. These cells were cultured in the culture medium containing 150 nM epirubicin (EPI, Fujifilm Wako Chemicals) to maintain the resistance to EPI.

The specific DDR2 inhibitor [31] WRG-28 was purchased from Sigma-Aldrich (St. Louis, MO, USA). Cell Matrix Type I-C (collagen I, 3 mg/mL) was purchased from Nitta Gelatin (Osaka, Japan), and the culture plates were coated with collagen I according to the manufacturer’s protocols before cell seeding.

### 2.5. Plasmid Construction, siRNAs, and Transfection

The full-length open reading frame of human DDR2 was amplified by PCR using KOD FX Neo (Toyobo, Osaka, Japan) and cloned into pcDNA3.1 (-) vector (Invitrogen Life Technologies Inc., Carlsbad, CA, USA) by ligation reaction using DNA ligation kit Mighty Mix (Takara Bio, Shiga, Japan). The plasmid was transfected into breast cancer cells using the Avalanche-Everyday Transfection Reagent (APRO Science, Tokushima, Japan). In addition, siRNAs targeting DDR2 (siDDR2-1, 2) were purchased from Thermo (Waltham, MA, USA). The sequence of siRNAs was as follows: siDDR2-1; GUAUGAGAGUGGAGCUUUAtt, siDDR2-2; CACUCCAUCUGGACAUUUAtt. MISSION siRNA Universal Negative Control (Sigma-Aldrich) was employed as a negative control (siNC).

### 2.6. Immunoblotting

Total protein was extracted using M-PER Mammalian Protein Extraction Reagent (Pierce Biotechnology, Rockford, IL, USA) containing Protease Inhibitor Cocktail (Sigma-Aldrich) according to the previous reports [32,33]. Protein extract (7.5 μg) was separated by SDS-PAGE (10% acrylamide gel) and transferred onto Hybond PVDF membranes (Fujifilm Wako Chemicals). The primary anti-DDR2 antibody used was the same as that used in immunohistochemistry. Anti-β-actin antibody (A3854, Sigma-Aldrich) was used as a loading control. The antibody–protein complex was visualized with ImmunoStar LD (Fujifilm Wako Chemicals) and LAS-4000 image analyzer (Fuji Photo Film Co., Tokyo, Japan).

### 2.7. RT-qPCR

Total RNA extraction was performed according to the previous report [34]. Briefly, total RNA was extracted using TRI reagent (Molecular Research Center, Cincinnati, OH, USA), and cDNA was synthesized using Rever Tra Ace qPCR RT Master Mix with gDNA Remover (Toyobo). A real-time PCR was performed using THUNDERBIRD SYBR qPCR Mix (Toyobo) and LightCycler nano system (Roche Diagnostics Japan, Tokyo, Japan). The DDR2 mRNA was normalized by the RPL13a mRNA level. The sequences of primers are as follows: DDR2; GGAGGTCATGGCATCGAGTT (forward) and GAGTGCCATCCCGACTGTAATT (reverse), RPL13a; CCTGGAGGAGAAGAGGAAAGAGA (forward) and TTGAGGACCTCTGTGTATTTGTCAA (reverse).

### 2.8. Cell Proliferation Assay

Breast cancer cells were transfected with DDR2 expression plasmids in advance, seeded into collagen-coated and non-collagen-coated 96-well culture plates (7500 cells/well), and followed to attach for 24 h. The cell proliferation was then evaluated using the Cell Counting Kit-8 (Dojindo, Molecular Technologies, Kumamoto, Japan) [35].

### 2.9. Caspase 3/7 Assay

The cells were transfected with either pCtrl or pDDR2 plasmid for 24 h and exposure by EPI for 72 h. Finally, apoptosis was evaluated using Caspase-Glo 3/7 Assay System (Promega, Madison, WI, USA). Relative Light Unit was measured by GloMax plate reader (Promega) and normalized by protein concentration to determine the relative caspase 3/7 activity.

### 2.10. Statistical Analysis

Statistical analyses were performed using JMP Pro 17 software (SAS Institute, Cary, NC, USA) according to manufacturer’s protocol. The association between DDR2 status or collagen type I and clinicopathological factors was evaluated using the Wilcoxon rank-sum test or $\chi^{2}$ test. Disease-free and breast cancer-specific survival curves were generated by the Kaplan–Meier method and assessed by the log-rank test. Univariate and multivariate analyses were evaluated using a proportional hazard model (Cox) and representative prognostic factors (pathological T factor (pT), lymph node metastasis, histological grade, ER, PR, and Ki67) were included. The parameters with a *p* value of <0.05 in the univariate analysis were subsequently analyzed in multivariate analysis.

For in vitro experiments, statistical significance was examined by Student’s *t*-test and Scheffe’s multiple comparisons using the StatView 5.0J software (SAS Institute, Cary, NC, USA) and data were presented as mean ± standard deviation (n = 4). Reproducibility was further confirmed by independent experiments. *p* < 0.05 was considered significant.

## 3. Results

### 3.1. Immunolocalization of DDR2 and Collagen Type I in Human Breast Cancer

**Figure 1:**
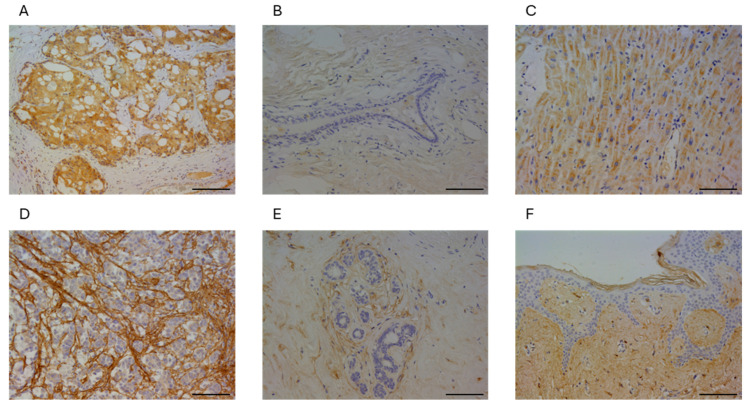
Representative image of DDR2 and collagen type I immunostaining in human breast cancer. (**A**–**C**): immunostaining of DDR2 in breast cancer cells (**A**), normal breast epithelium (**B**), and human heart as a positive control of DDR2 (**C**). (**D**–**F**): immunostaining of collagen type I in cancerous stroma (**D**), normal breast stroma (**E**), and human skin as a positive control of collagen type I (**F**). Bar = 100 µm, respectively.

We first immunolocalized DDR2 and collagen type I in 224 breast cancer specimens, and the immunoreactivity of DDR2 was observed in the cytoplasm of the carcinoma cells (Figure 1A), while it was quite slight in normal breast epithelium (Figure 1B). Collagen type I immunoreactivity was predominantly observed in the ECM of cancer stroma (Figure 1D) compared to the ECM surrounding normal breast epithelium (Figure 1E). In the positive control of immunostaining, DDR2 immunoreactivity was observed in the myocardium (Figure 1C), while that of collagen type I was observed in the dermis (Figure 1F).

The correlation between DDR2 or collagen type I immunoreactivity and clinicopathological parameters in breast cancer is summarized in Table 1. The number of DDR2-positive cases was 75 out of 224 (33.5%) and that of collagen type I-positive cases was 138 out of 224 (61.6%). DDR2 immunoreactivity was positively associated with histological grade (*p* = 0.0012) and Ki67 LI (*p* = 0.0004). DDR2 immunoreactivity was more frequently observed in the breast cancer tissues following neoadjuvant chemotherapy (*p* = 0.010). On the other hand, collagen type I immunoreactivity was positively associated with lymph node metastasis (*p* = 0.039) and inversely associated with Ki67 LI (*p* = 0.0076). Furthermore, collagen type I immunoreactivity was more frequently observed in the breast cancer tissues following neoadjuvant chemotherapy (*p* = 0.013).

As shown in Table 2, when we associated DDR2 immunoreactivity according to collagen type I status, DDR2 immunoreactivity was positively associated with pathological T factor (pT) (*p* = 0.017) and Ki67 LI (*p* = 0.033), while it was negatively associated with PR status (*p* = 0.012) only in the collagen type I-positive group. In addition, the patients positive for both DDR2 and collagen type I showed higher pT, higher stage, and PR negativity (Supplementary Table S1).

### 3.2. Correlation Between DDR2 and Collagen Type I Immunoreactivity and Clinical Outcome of Breast Cancer Patients

**Figure 2:**
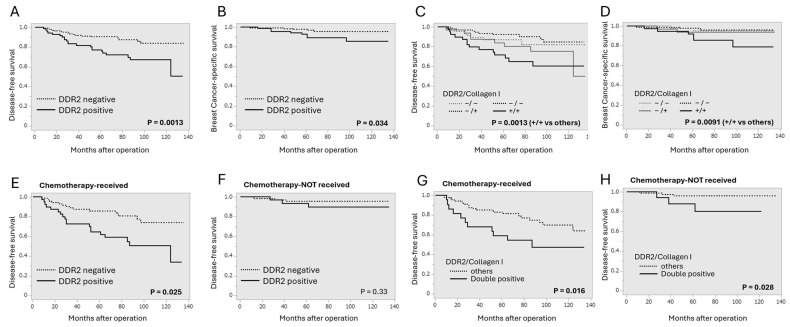
Association between DDR2 status and clinical outcomes of breast cancer patients (n = 224). (**A**–**D**): disease-free survival (**A**,**C**) and breast cancer-specific survival (**B**,**D**) according to DDR2 status (**A**,**B**) or DDR2/collagen type I combination status (**C**,**D**). (**E**–**H**): disease-free survival according to DDR2 status (**E**,**F**) or DDR2/collagen type I combination status (**G**,**H**) in the patients who received chemotherapy (**E**,**G**) or not (**F**,**H**).

We next analyzed the association between DDR2 or collagen type I status and clinical outcomes of breast cancer patients (Figure 2). DDR2 status was significantly associated with shorter disease-free survival (*p* = 0.0013, Figure 2A) and shorter breast cancer-specific survival (*p* = 0.034, Figure 2B). When we examined the prognostic significance of DDR2 status according to collagen type I status, the DDR2/collagen type I double-positive phenotype was significantly associated with adverse clinical outcomes (Figure 2C,D). Interestingly, DDR2 status was associated with shorter disease-free survival in the patients who received chemotherapy (Figure 2E) but not in those without chemotherapy (Figure 2F). The DDR2/collagen type I double-positive phenotype was associated with shorter disease-free survival regardless of chemotherapy (Figure 2G,H), but it seemed to be correlated more strongly in the patients with chemotherapy.

Next, we confirmed the prognostic implication of DDR2 by univariate and multivariate analysis (Table 3). In the univariate analysis, DDR2 status (*p* = 0.0019) as well as pT (*p* < 0.0001), lymph node metastasis (*p* = 0.032), histological grade (*p* = 0.032), ER (*p* = 0.012), PR (*p* = 0.0006), and Ki67 LI (*p* = 0.0002) were demonstrated as significant prognosis factors. The following multivariate analysis revealed that DDR2 status (*p* = 0.0095) was identified as an independent prognostic factor for the recurrence of breast cancer along with pT (*p* = 0.012) and Ki67 LI (*p* = 0.031). Importantly, DDR2 status was also an independent prognostic factor for disease-free survival in the patients who received chemotherapy.

### 3.3. Effect of DDR-2 on the Proliferation of Breast Cancer Cells

**Figure 3:**
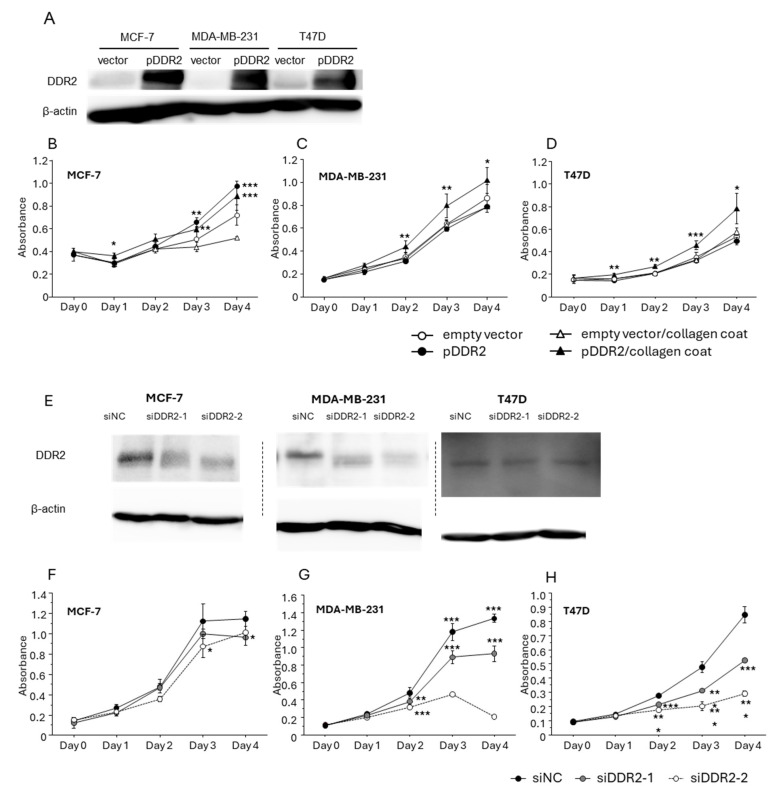
The effect of DDR2 in the proliferation of human breast cancer cell lines in the presence of collagen type I. (**A**) Immunoblotting of exogenous DDR2 protein in MCF-7, MDA-MB-231, and T47D cells. (**B**–**D**): cell proliferation of MCF-7 (**A**), MDA-MB-231 (**B**), and T47D (**D**) transfected with an empty vector or DDR2-expressing vector in the absence or presence of collagen coating. (**E**) Immunoblotting of DDR2 in the cells transfected with siRNA against DDR2 (siDDR2-1, 2). (**F**–**H**): cell proliferation of MCF-7 (**F**), MDA-MB-231 (**G**), and T47D (**H**) transfected with siRNAs in the presence of collagen coating. * *p* < 0.05, ** *p* < 0.01, and *** *p* < 0.001 compared to the empty vector, respectively.

Since we observed a significant association between DDR2/collagen type I and the proliferative ability of breast cancer in immunohistochemical analysis, we performed the cell proliferation assay using human breast cancer cell lines. Firstly, the cells were transfected with DDR2-expressing plasmid and a significant level of DDR2 protein induction was confirmed (Figure 3A). The proliferation of MCF-7 (Figure 3B) was significantly increased by exogenous DDR2 expression independently of collagen coating. On the other hand, the proliferation of MDA-MB-231 (Figure 3C) and T47D (Figure 3D) were significantly increased by exogenous DDR2 expression in the presence of collagen type I. Next, the cells were also transfected with siRNAs targeting DDR2 (siDDR2-1, 2) to suppress the endogenous DDR2 (Figure 3E). Importantly, the proliferation of MCF-7 (Figure 3F), MDA-MB-231 (Figure 3G), and T47D (Figure 3H) was significantly suppressed by knockdown of DDR2 in the presence of collagen I.

### 3.4. Increased DDR2 Expression in EPI-Resistant Breast Cancer Cells and the Effect of DDR2 on Chemotherapy Resistance in Breast Cancer Cells

**Figure 4:**
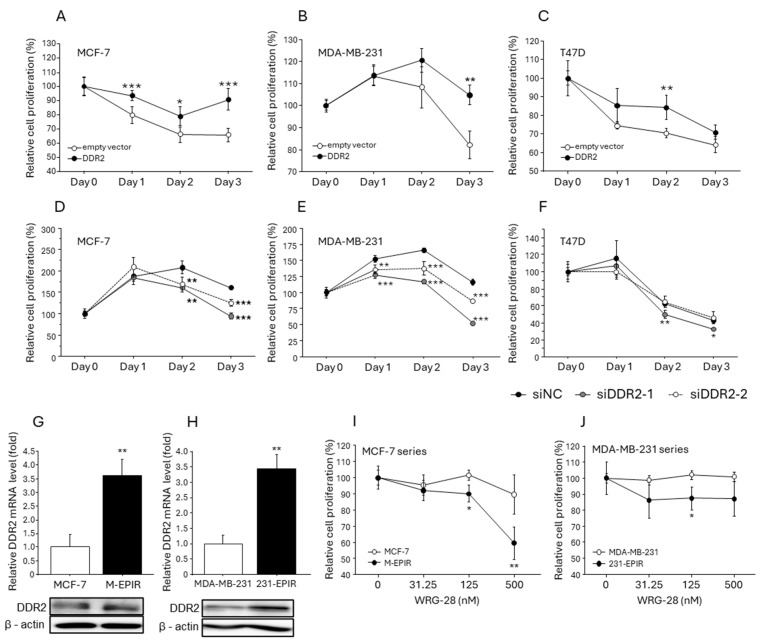
The effect of DDR2 on the resistance to epirubicin in the breast cancer cell lines. (**A**–**C**): viability of MCF-7 (**A**), MDA-MB-231 (**B**), and T47D (**C**) transfected with an empty vector or DDR2-expressing vector under epirubicin treatment (500 nM for MCF-7; 250 nM for MDA-MB-231 and T47D). These cells were plated onto collagen-coated culture plates. * *p* < 0.05, ** *p* < 0.01, and *** *p* < 0.001 compared to the empty vector, respectively. (**D**–**F**): viability of MCF-7 (**D**), MDA-MB-231 (**E**), and T47D (**F**) transfected with siRNA targeting DDR2 under epirubicin treatment. (**G**,**H**) mRNA and protein expression in chemosensitive parental cells and epirubicin-resistant cells (**G**; MCF-7 series, **H**; MDA-MB-231 series). ** *p* < 0.01, respectively. (**I**,**J**) The effect of DDR2 inhibitor WRG-28 treatment (48 h) on the proliferation of chemosensitive parental cells and epirubicin-resistant cells (**F**; MCF-7 series, **G**; MDA-MB-231 series).

Since our immunohistochemical analysis demonstrated that DDR2 was associated with chemotherapy resistance in breast cancer, we subsequently verified the possible contribution of DDR2 to breast cancer chemoresistance using both chemoresistant breast cancer cell lines (M-EPIR and 231-EPIR) and chemosensitive (parental) breast cancer cell lines. We confirmed that the proliferation of MCF-7 (Figure 4A), MDA-MB-231 (Figure 4B), and T47D (Figure 4C) in the presence of EPI was significantly maintained by exogenous DDR2 expression. On the contrary, cell proliferation was more strongly suppressed by EPI in the cells transfected with siRNA for DDR2 (Figure 4D–F). Interestingly, the endogenous expression levels of DDR2 were elevated in both mRNA and protein levels in M-EPIR and 231-EPIR cells compared to parental cells (Figure 4G,H). To elucidate the role of DDR2 in the proliferation of EPI-resistant cell lines, they were treated with the specific DDR2 inhibitor WRG-28, and the cell proliferation was significantly inhibited by WRG-28 in M-EPIR and 231-EPIR, while it was not significantly changed in parental MCF-7 and MDA-MB-231 cells. We confirmed that both M-EPIR and 231-EPIR cells were sensitive to the DDR2-specific inhibitor, WRG-28 (Figure 4I,J).

### 3.5. Effect of DDR-2 on the Apoptosis of Breast Cancer Cells

**Figure 5:**
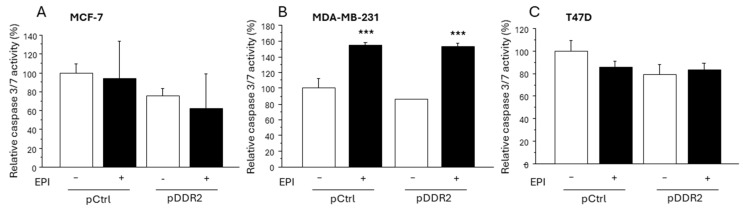
The effect of DDR2 on the apoptosis of breast MCF-7 (**A**), MDA-MB-231 (**B**), and T47D (**C**). *** *p* < 0.001.

Since chemotherapy often induces the apoptosis of breast cancer cells, we examined the effect of DDR2 on the apoptosis of breast cancer cells. EPI did not induce apoptosis in MCF-7 (Figure 5A) and T47D (Figure 5C) cells transfected with both the pCtrl group and the pDDR2-transfected group. On the other hand, EPI significantly induced apoptosis in MDA-MB-231 cells regardless of the exogenous DDR2 expression status (Figure 5B).

## 4. Discussion

Growing evidence has suggested that ECM composition is markedly altered in breast cancer compared to the normal mammary glands and that the cancer cell–ECM interaction has a pivotal role in breast cancer progression [36]. Collagen type I is a representative fibrillar collagen highly deposited in human malignancies including breast cancer [37] and transduces protumor signals in breast cancer cells via its receptors such as DDR2. To the best of our knowledge, this is the first study that comprehensively examined the significance of DDR2 and collagen type I. When we immunolocalized DDR2 and collagen type I in 224 human breast cancer tissues, their immunoreactivity was significantly increased in carcinoma cells or cancerous stroma compared to normal breast epithelium or stroma. Although the mechanisms of aberrant DDR2 expression in breast cancer are yet to be fully elucidated, epithelial–mesenchymal transition (EMT)-associated transcription factors such as TWIST and SNAIL are reported to induce DDR2 in breast cancer [38]. In addition, dysregulated methylation or STAT3 activation is also associated with DDR2 expression in gastric cancer [39], epithelial ovarian cancer [15], and hepatocellular carcinoma [14]. These findings might suggest the pivotal roles of DDR2 in breast cancer progression. Of importance, DDR2 immunoreactivity was positively associated with the aggressive behavior of breast cancers such as a higher invasion ability (pT) and cell proliferation (Ki67), especially in the collagen type I-positive group. In addition, exogenous DDR2 expression significantly increased the cell proliferation of MDA-MB-231 and T47D cells in the presence of collagen type I coating, while knockdown of DDR2 suppressed the proliferation of these cells. These findings suggest that DDR2 promotes breast cancer cell proliferation and the invasion of breast cancer cells in a collagen type I-dependent fashion, which is consistent with a previous report demonstrating the collagen-dependent pro-proliferative effect of DDR2 in gastric cancer [39]. On the other hand, we found that DDR2 promoted the cell proliferation of MCF-7 regardless of collagen type I. This may arise from the fact that MCF-7 itself can produce collagen protein [40] and endogenous collagens may be able to activate DDR2.

Therapeutic resistance, especially chemoresistance, is one of the most important issues to be solved in breast cancer. Our recent prognosis analysis revealed that DDR2 immunoreactivity was associated with adverse clinical outcomes in breast cancer, like the previous report [11,26,41]. However, it should be noted that our present analysis demonstrated that DDR2 and collagen type I immunoreactivity was more frequently observed in the breast cancer tissues treated with neoadjuvant chemotherapy, while DDR2 contributed to breast cancer recurrence cooperatively with collagen type I and served as a strong prognostic factor, especially in those who received chemotherapy. These findings suggest a possible relationship between DDR2 and the chemoresistance of breast cancer. In vitro experiments also demonstrated that overexpression of DDR2 significantly increased cell viability in the EPI-treated MCF-7 and MDA-MB-231 cells in the presence of collagen type I coating, while knockdown of DDR2 significantly restored the chemosensitivity to EPI. On the contrary, DDR2 inhibitor WRG-28 suppressed the cell proliferation of EPI-resistant breast cancer cell lines (M-EPIR and 231-EPIR), in which DDR2 expression was significantly increased compared to parental cell lines. It is therefore reasonably hypothesized that DDR2 and increased collagen deposition is associated with the acquired chemoresistance of breast cancer. It has been proposed that the DDR2/collagen axis promotes EMT in human cancers including breast cancer [42,43], while EMT is well known to be associated with the chemoresistance of breast cancer [44,45]. Collagen type I has been reported to induce breast cancer chemoresistance by activating ABC efflux transporters via another collagen type I-binding protein, integrin β1 [46]. Altered ECM environments following chemotherapy should be receiving attention for a better understanding of the mechanisms of breast cancer chemoresistance and exploring novel biomarkers to improve the treatment. On the other hand, exogenous DDR2 expression did not affect the apoptosis induced by EPI. It is therefore hypothesized that DDR2 contributed to breast cancer chemoresistance independently of the apoptosis of breast cancer cells.

## 5. Conclusions

We demonstrated in the present study that DDR2 contributes to breast cancer chemoresistance cooperatively with collagen type I and serves as a potent prognostic factor in breast cancer patients receiving chemotherapy.

## Figures and Tables

**Table 1:** Association between DDR2 status and clinicopathological factors in 224 breast carcinomas.

	DDR2			Collagen Type I		
	Negative (n = 149)	Positive (n = 75)	*p*	Negative (n = 86)	Positive (n = 138)	*p*
Age *	55 (31–88)	56 (27–82)	0.86	55 (29–88)	55 (27–85)	0.39
pT						
pT1	109	46		65	90	
pT2–4	40	29	0.071	21	48	0.10
Lmph node metastasis						
Negative	100	51		65	86	
Positive	49	24	0.89	21	52	**0.039**
Stage						
I	90	38		56	72	
II	38	23		21	40	
III	21	14	0.37	9	26	0.11
Histological grade						
1	67	15		27	55	
2	56	42		37	61	
3	26	18	**0.0012**	22	22	0.17
ER						
Negative	25	16		20	21	
Positive	124	59	0.41	66	117	0.13
PR						
Negative	42	28		29	41	
Positive	107	47	0.16	57	97	0.53
HER2						
Negative	128	61		70	119	
Positive	21	14	0.37	17	19	0.33
Ki67 LI (%) *	10 (1–60)	17 (1–72)	**0.0004**	14 (2–53)	12 (1–72)	**0.0076**
Neoadjuvant chemotherapy						
Not received	129	55		77	107	
Received	19	20	**0.010**	8	31	**0.013**
Collagen I						
Negative	51	35				
Positive	98	40	0.071			

* Data were presented as the median (minimum–max). *p*-value < 0.05 was significant (in bold).

**Table 2:** Association between DDR2 status and clinicopathological factors according to collagen type I status in 224 breast carcinomas.

	Collagen I-Negative Group			Collagen I-Positive Group		
	DDR2			DDR2		
	Negative (n = 51))	Positive (n = 35)	*p*	Negative (n = 124)	Positive (n = 59)	*p*
Age *	56 (31–88)	58 (29–82)	0.46	55 (33–85)	55 (27–77)	0.26
pT						
pT1	39	26		70	20	
pT2–4	12	9	0.82	28	20	**0.017**
Lymph node metastasis						
Negative	38	27		62	24	
Positive	13	8	0.78	36	16	0.72
Stage						
I	34	22		56	16	
II	14	7		24	16	
III	3	6	0.22	18	8	0.14
Histological grade						
1	21	6		46	9	
2	16	21		40	21	
3	14	8	**0.019**	12	10	**0.018**
ER						
Negative	12	8		13	8	
Positive	39	27	0.94	85	32	0.32
PR						
Negative	19	10		23	18	
Positive	32	25	0.40	75	22	**0.012**
HER2						
Negative	44	26		84	35	
Positive	7	9	0.16	14	5	0.78
Ki67 LI (%) *	12 (2–52)	16 (3–53)	0.12	9 (1–60)	18 (1–72)	**0.0033**

* Data were presented as the median (minimum–max). *p*-value < 0.05 was significant (in bold).

**Table 3:** Uni- and multivariate analysis of disease-free survival.

	All Cases			Chemotherapy-Received Group		
	Uni-	Multi-		Uni-	Multi-	
	*p*	*p*	CI	*p*	*p*	CI
pT	<0.0001 *	**0.012**	**2.7 (1.2–5.7)**	0.063		
pT2–4/pT1						
Lymph node metastasis	0.032 *	0.31	1.5 (0.70–3.1)	0.87		
Positive/Negative						
Histological grade	0.032 *	0.26	0.60 (0.24–1.5)	0.81		
3/1+2						
ER	0.012 *	0.82	1.1 (0.42–3.0)	0.38		
Negative/Positive						
PR	0.0006 *	0.069	2.1 (0.94–4.9)	0.049 *	**0.0004**	**3.7 (1.8–7.8)**
Negative/Positive						
HER2	0.13			0.032 *	**0.0036**	**0.16 (0.048–0.55)**
Positive/Negative						
Ki67	0.0002 *	**0.031**	**2.4 (1.1–5.3)**	0.072		
≤20/<20						
DDR2	0.0019 *	**0.0095**	**2.4 (1.2–4.7)**	0.0038 *	**0.0033**	**2.8 (1.4–5.7)**
Positive/Negative						
Collagen type I	0.94			0.75		
Positive/Negative						

* *p* < 0.05 was considered significant and incorporated in multivariate analysis. CI: 95% confidence interval. *p*-value < 0.05 was significant (in bold).

## Data Availability

The original contributions presented in this study are included in the article/Supplementary Materials. Further inquiries can be directed to the corresponding author.

## Supplementary Materials

The following supporting information can be downloaded at https://www.mdpi.com/article/10.3390/cancers16244285/s1: Table S1: Association between immunohistochemical DDR2/collagen type I status and clinicopathological factors in 224 breast carcinomas.
