# Supplementary material for: Discoidin Domain Receptor 2 Contributes to Breast Cancer Progression and Chemoresistance by Interacting with Collagen Type I
Source: Cancers (Basel). 2024 Dec 23;16(24):4285. doi: 10.3390/cancers16244285 (PMC11674238; doi:10.3390/cancers16244285)
Supplement: Supplementary file 1 [file cancers-16-04285-s001.zip › cancers-3395844-supplementary.pdf]

Supplementary Table S1. Association between immunohistochemical DDR2/collagen type I status and clinicopathological factors in 224 breast carcinomas.

	DDR2/collagen type I		
	Others	Double Positive	P
Age	56 (29-88)	55 (27-77)	0.19
Menopausal status			
Pre-	67	18	
Post	117	22	0.31
pT			
pT1	135	20	
pT2-4	49	20	**0.0037**
Lymph node metastasis			
Negative	127	24	
Positive	57	16	0.27
Stage			
I	112	16	
II	45	16	
III	27	8	**0.049**
Histological grade			
1	73	9	
2	77	21	
3	34	10	0.12
ER			
Negative	33	8	
Positive	151	32	0.76
PR			
Negative	52	18	
Positive	132	22	**0.038**
HER2			
Negative	154	35	
Positive	30	5	0.55
Ki67 labeling index (LI; %)	12 (1-60)	18 (1-72)	0.092
Neoadjuvant chemotherapy			
Not received	77	107	
Received	8	31	**0.013**
